# TRPV1 in dorsal root ganglion contributed to bone cancer pain

**DOI:** 10.3389/fpain.2022.1022022

**Published:** 2022-11-09

**Authors:** Wen Chen, Hongping Li, Xiaowan Hao, Cunzhi Liu

**Affiliations:** ^1^ International Acupuncture and Moxibustion Innovation Institute, Beijing University of Chinese Medicine, Beijing, China; ^2^ School of Acupuncture-Moxibustion and Tuina, Beijing University of Chinese Medicine, Beijing, China

**Keywords:** bone cancer pain, dorsal root ganglion, TRPV1, regulation, mechanisms

## Abstract

Tumor growth *in situ* or bone metastases in cancer patients all can induce bone cancer pain. It is frequently occurred in patients with breast, prostate, and lung cancer. Because of the lack of effective treatment, bone cancer pain causes depression, anxiety, fatigue, and sleep disturbances in cancer patients, disrupts the daily quality of life, and results in huge economic and psychological burden. Over the past years, transient receptor potential channels (TRPs), especially TRP vanilloid 1 (TRPV1) in dorsal root ganglion (DRG), have been considered to be involved in bone cancer pain. The characteristic of TRPV1 had been well studied. The mechanisms under TRPV1 regulation in DRG with bone cancer pain are complex, including inflammatory mediators, endogenous formaldehyde, and other mechanisms. In the present review, we summarize the role and potential mechanism of TRPV1 in DRG in bone cancer pain. As the primary sensory neurons, targeting the TRPV1 channel in DRG, might have fewer side effects than in central. We hope systematically understand of TRPV1 modulation in DRG will bring more effective strategy.

## Literature selection

We searched on PubMed with the keyword [“Bone cancer pain” or “Cancer-associated bone pain” and “transient receptor potential vanilloid subfamily member 1 or TRPV1”] or [“Bone cancer pain” or “Cancer-associated bone pain” and “transient receptor potential vanilloid subfamily member 1 or TRPV1” and “dorsal root ganglion or DRG”]. A total of 58 articles were retrieved, because of the absence of full text, 6 articles were excluded. Next, we excluded 24 articles based on title or abstract, including 4 reviews and 20 articles, that had very little to the subject. On the other hand, we added one article that related to the definition of bone cancer pain, two articles that related to the characteristics of TRPV1, two articles related to the expression profile of TRPV1 in DRG, one article about the regulation of TRPV1 by TNF, two articles, and one review about PD-L1. Finally, 37 articles were included, containing 27 basic research articles and 10 reviews.

## TRPV1 channel

In 1997, transient receptor potential vanilloid subfamily member 1 (TRPV1) was first described as the receptor of capsaicin (1). Since then, more and more researchers gave their attention to the TRPV1 channel (2–4). In addition to capsaicin, TRPV1 also can be activated by other factors, including heat, protons, resiniferatoxin (RTX, found in the latex of *Eucalyptus resinifera*), eugenol (a compound present in cloves), allicin (found in garlic), piperine (an irritant found in black pepper), gingerol and zingerone (from ginger), camphor (found in the wood of camphor), and the hot component of pepper (1, 5–8). In bone cancer pain, the acidic microenvironment could activate the TRPV1 channel although the expression of TRPV1 mRNA had no change (9). TRPV1 is broadly expressed in peripheral nervous system and the abnormal activity of TRPV1 is involved in a variety of pathophysiological processes, such as pain and itch. In DRG neurons, TRPV1 is predominantly located in small and medium neurons (10–12), the distribution of TRPV1 is consistent with the heat sensitivity of TRPV1. The bone cancer pain model led to an overall shift in the distribution of TRPV1 from small to large neurons, validating that large DRG neurons expressed TRPV1 (10, 11). In the bone cancer pain model, the TRPV1 channel contributes to the sensitization of DRG neurons (13, 14).

## TRPV1 contributed to bone cancer pain

Bone cancer pain can be classified into primary cancer pain or secondary cancer pain (15, 16). Due to the complexity and not fully understand the mechanism (17), resulted in the effective treatment of bone cancer pain was blocked. In 2005, TRPV1 was first demonstrated to be involved in bone cancer pain (18). In this study, bone cancer pain was induced by the injection of 2,472 osteolytic sarcoma cells to the mouse femur. Resulted in a large proportion of sensory neurons innervating the tumor-bearing bone expressed TRPV1. Subcutaneous injection of TRPV1 antagonist JNJ-17203212 alleviated pain behaviors at different stages of cancer progression. Although the antagonist of TRPV1 attenuated the pain behavior, blockade TRPV1 not always reduced bone cancer pain indicating that the regulation of TRPV1 was multiple (19–21). Furthermore, the TRPV1^+/+^ and TRPV1^+/−^ animals had normal development of pain behaviors, whereas TRPV^−/−^ mice showed a reduction in the development of behaviors. In another study, Walker 256 cells were injected into the bone cavity of rat tibial to induce bone cancer pain. The rats developed mechanical allodynia and thermal hyperalgesia. Intrathecal injection of adeno-associated virus (AAV) mediated siRNA against TRPV1 significantly suppressed the expression of *TRPV1* mRNA in DRG and alleviated the pain behavior of rats (7). Upregulation of membrane TRPV1 in DRG was also found in rats with bone cancer pain. In the study, the animal model was induced by injection of mammary rat metastasis tumor (MRMT-1) cells (carcinoma) into tibial bone cavity. Not only that, the current density of TRPV1 was significantly increased in DRG neurons (14). All the studies suggested that TRPV1 in DRG was upregulated and involved in bone cancer pain. But we also noted that there are some contradictions in the expression of TRPV1. A study showed that the expression of TRPV1 in DRG was decreased in bone cancer, but capsazepine, an another antagonist of TRPV1, significantly alleviated the pain behavior by subcutaneous injection (22). In another research, the expression of TRPV1 mRNA had no change in DRG (9). Different experimental conditions may affect the expression of TRPV1. In addition to experimental conditions, membrane TRPV1 plays more important role in bone cancer pain. The activity of TRPV1 could increase when the expression had no change and involved in cancer pain. The decreased expression of TRPV1 did not necessitate their decreased activity in neuron membrane (22).

## Mechanisms of TRPV1 regulation in DRG of bone cancer pain

### Inflammatory mediators

TRPV1 receptor can be regulated by inflammatory mediators, including interleukin-6 (IL-6), tumor necrosis factor-α (TNF-α), insulin-like growth factor-1 (IGF-1), and so on. In cultured DRG neurons, IL-6 treatment significantly upregulated the membrane protein level and current density of TRPV1. Additionally, the suppression of the IL-6 signaling by sgp130 reversed the phenomena and companied with bone cancer pain behavior relief in rats (14). Administration of TNF-α to cultured DRG neurons, the protein level and current density of TRPV1 were all both upregulated. Furthermore, the study found that TRPV1 protein level in DRG was increased in wild-type mice but not in TNFR2^−/−^ mice with tumor (23). In DRG neurons, IGF-1 receptor co-expressed with TRPV1. Cultured DRG neurons with IGF-1, increased the total and membrane protein level of TRPV1. Inhibition of IGF-1 receptor with picropodophyllotoxin alleviated the pain behavior (24, 25). These results suggested that IGF-1 might regulate the expression of TRPV1 and contribute to bone cancer pain.

In the bone cancer pain model, tumor growth disrupted the balance between osteoclast and osteoblast in bone (26). On the one hand, cancer cells not only promoted osteoclasts secretion of proton, but also inflammatory mediators (27), on the other hand, cancer cells also can secret inflammatory mediators and other substance (28). These substances can activate or upregulate TRPV1 and then contribute to the sensitization of sensory nervous in pain signaling transmission.

### Endogenous formaldehyde

The local microenvironment in the bone is complex. In the development of cancer, tumor tissues can secrete formaldehyde. In cultured cancer cell lines, such as MRMT-1 cells, H1299 cells, SY5Y cells, and Walker 256 cells, the formaldehyde concentration was significantly increased. The same results could be observed in tumor tissues from cancer patients (29). Formaldehyde treatment increased the expression and current density of TRPV1 (29, 30). These results suggested in bone cancer pain models, formaldehyde upregulated the expression of TRPV1 and contributed to pain behavior. The fact suggested that target the formaldehyde production might be a potential treatment for bone cancer pain.

### Other mechanisms

It is generally believed that cancer pain is complex, so the regulation mechanisms of TRPV1 are also complex. In addition to the above mentioned, there are other mechanisms. In the early phase of cancer, patients feel no pain or minimal pain may be due to the antinociceptive effects of programmed death ligand 1 (PD-L1) (31–33). In a study, Liu and colleagues found the expression of PD-1, receptor of PD-L1, was also increased. PD-L1 activated Src homology 2 domain-containing tyrosine phosphatase-1 (SHP-1) and inhibited the expression of TRPV1 in DRG. Besides, PD-L1-induced analgesia on bone cancer pain was only observed in wild-type but not in TRPV1-KO mice (34). High mobility group box 1 (HMGB1) is one of the damage-associated molecular patterns. There was a report indicated that HMGB1 contributed to bone cancer pain by upregulation of TRPV1, but the behind mechanism had not been fully illustrated (35). Lysophosphatidic acid (LPA) is a lipid metabolite released after tissue injury, and plays a key role in cancer development. In bone cancer pain rats, LPA potentiated TRPV1 current *via* a protein kinase C (PKC)-dependent pathway in DRG neurons (36).

## Conclusion

In this mini review, we summarized the role and potential mechanism of DRG TRPV1 in bone cancer pain, including inflammatory mediators, endogenous formaldehyde, and other mechanisms. Although the mechanisms about TRPV1 regulation had been studied, target TRPV1 for bone cancer pain treatment is still limited because of the side effect. The mechanisms of pain include the peripheral and center nervous systems, when compared to the central, the structure of the peripheral is simpler. In the future, target TRPV1 in DRG might be a better choice for bone cancer pain treatment (37). In order to find more specific drug that target TRPV1, further research is still needed.
